# Molecular Mechanism of Z α1-Antitrypsin Deficiency*

**DOI:** 10.1074/jbc.M116.727826

**Published:** 2016-05-31

**Authors:** Xin Huang, Ying Zheng, Fei Zhang, Zhenquan Wei, Yugang Wang, Robin W. Carrell, Randy J. Read, Guo-Qiang Chen, Aiwu Zhou

**Affiliations:** From the ‡ Institute of Health Sciences, Shanghai Institutes for Biological Sciences, Chinese Academy of Sciences and Shanghai Jiao Tong University School of Medicine and; § University of Chinese Academy of Sciences, Shanghai 200025, China,; the ¶ Hongqiao International Institute of Medicine, Shanghai Tongren Hospital/Faculty of Basic Medicine, Chemical Biology Division of Shanghai Universities E-Institutes, Key Laboratory of Cell Differentiation and Apoptosis of the Chinese Ministry of Education, Shanghai Jiao Tong University School of Medicine, Shanghai 200025, China, and; the ‖ Department of Haematology, Cambridge Institute for Medical Research, University of Cambridge, Cambridge CB2 0XY, United Kingdom

**Keywords:** conformational change, crystal structure, protein folding, serpin, small molecule, PBA, Polymerization, Z α1-antitrypsin

## Abstract

The Z mutation (E342K) of α1-antitrypsin (α1-AT), carried by 4% of Northern Europeans, predisposes to early onset of emphysema due to decreased functional α1-AT in the lung and to liver cirrhosis due to accumulation of polymers in hepatocytes. However, it remains unclear why the Z mutation causes intracellular polymerization of nascent Z α1-AT and why 15% of the expressed Z α1-AT is secreted into circulation as functional, but polymerogenic, monomers. Here, we solve the crystal structure of the Z-monomer and have engineered replacements to assess the conformational role of residue Glu-342 in α1-AT. The results reveal that Z α1-AT has a labile strand 5 of the central β-sheet A (s5A) with a consequent equilibrium between a native inhibitory conformation, as in its crystal structure here, and an aberrant conformation with s5A only partially incorporated into the central β-sheet. This aberrant conformation, induced by the loss of interactions from the Glu-342 side chain, explains why Z α1-AT is prone to polymerization and readily binds to a 6-mer peptide, and it supports that annealing of s5A into the central β-sheet is a crucial step in the serpins' metastable conformational formation. The demonstration that the aberrant conformation can be rectified through stabilization of the labile s5A by binding of a small molecule opens a potential therapeutic approach for Z α1-AT deficiency.

## Introduction

**FIGURE 1.**
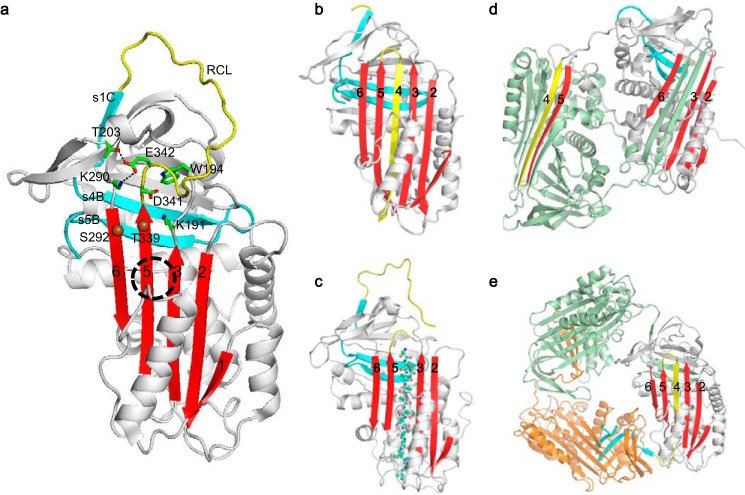
**Conformations of α1-AT.** *a*, metastable conformation of wild type α1-AT. Glu-342, located at the base of the reactive center loop (*yellow*), forms hydrogen bonds with Thr-203 and Lys-290. The so-called shutter region, which opens during reactive loop insertion, is *circled* by *dashed lines*. Atoms Cα of residue 229 and 339 for an engineered disulfide bond are shown as *spheres*. Trp-194 below the hinge region and surrounding residues Asp-341 and Lys-191 are shown as *sticks*. Once the reactive loop (*yellow*) is cleaved by protease, it is inserted into the central β-sheet (*red*) as a middle strand forming a hyperstable conformation (*b*). The peptide which encodes sequence from the serpin-reactive loop could also insert into the central β-sheet as a middle strand converting serpin into a hyper-stable binary complex conformation (*c*). Polymers may form though s4/5A domain swap linkage derived from antithrombin dimer structure (*d*) and the C-terminal s4/5B (*cyan*) linkages derived from crystal structure of α1-AT trimer (*e*).

Serpins (1–3) are folded into a metastable conformation with a surface-exposed reactive center loop (Fig. 1*a*). Once the reactive loop is recognized and cleaved by the target protease, a dramatic conformational change occurs, with the incorporation of the reactive loop into the middle of the central β-sheet A and a translocation and inactivation of the covalently linked protease (4–6). This unique change from a metastable to hyperstable conformation (Fig. 1*b*) is accompanied by a large free energy change, which is utilized for protease inhibition.

However, the native metastable conformation is susceptible to point mutations that cause serpin misfolding and polymerization and subsequent retention of stable serpin polymers within the endoplasmic reticulum of cells (7, 8). The Z mutation (E342K) of α1-antitrypsin (α1-AT),^4^ carried by 4% of Northern Europeans, predisposes to the early onset of emphysema due to decreased functional α1-AT in the lung and to liver cirrhosis due to accumulation of α1-AT polymers in the endoplasmic reticulum of hepatocytes (9–12). Glu-342 is located at the top of strand 5 of central β-sheet A (s5A) in the P17 position (active site residue termed P1) at the base of the reactive center loop and forms a highly conserved salt bridge to Lys-290 and a hydrogen bond to Thr-203 (Fig. 1*a*). This together with main chain packing effectively forces the reactive loop to take a turn with residue 342 acting as a hinge (13).

The mechanism by which the E342K mutation causes polymerization of Z α1-AT is not fully understood, and various models of serpin polymerization have been proposed (12, 14–18). The “classic” loop-sheet model proposed that serpin polymers could form by the intermolecular linkage of the reactive loop of one molecule with the β-sheet A of another (12, 14). Similarly a β-hairpin model was proposed based on the crystal structure of an antithrombin dimer (Fig. 1*d*) with a larger domain swap, including the reactive loop and strand 5 of the central β-sheet A (s5A) (16), but subsequent immunological evidence challenged this as the linkage that occurs *in vivo* (19, 20). A later serpin polymerization model, derived from the crystal structure of an α1-AT trimer (17) (Fig. 1*e*), proposes that polymerization *in vivo* occurs through a C-terminal domain swap mechanism involving strands 4 and 5 of β-sheet B (s4/5B). However, these models do not satisfyingly explain how exactly the mutation of Glu-342 affects the folding pathway of α1-AT leading to polymerization, and the folding pathway of α1-AT proposed from the trimer structure (17) contradicts a subsequent model derived from biochemical studies (21, 22). Also unexplained is the finding that ∼15% of the expressed Z α1-AT is secreted into the plasma as an active, but unstable, monomer. This circulating Z α1-AT seems to adopt an aberrant conformation with a high basal fluorescence signal (23), which preferentially binds to a 6-mer peptide (FLEAIG) derived from its reactive center loop (23).

Here, we have assessed the role of residue 342 in α1-AT and solved the crystal structure of the Z α1-AT monomer. Our findings reveal how the mutation of Glu-342 would lead to an aberrant conformation of Z α1-AT and explain how the Z mutation will disrupt a key step in the folding pathway of α1-AT leading to its pathological polymerization.

## Results

### 

#### 

##### Role of Residue 342 in α1-AT Folding

The Z mutation (E342K) will not only result in the direct loss of the stabilizing interactions of Glu-342 but will also perturb the nearby packing due to the positive charge of the lysine side chain. However, there is no consensus as to which is the main contributing factor (24–27). To dissect this, we systematically mutated Glu-342 to the 19 other common amino acids and expressed these variants in a bacterial expression system that eliminates the effects of glycosylation and chaperone on folding as seen in mammalian cells. All the variants mentioned in this paper are based on the well documented Pittsburgh variant of α1-AT with an Arg at the P1 position (28) for convenient assessment of conformational change effects toward protease inhibition, and α1-AT-Pittsburgh is termed wild type here. We then compared the levels of overall expression of α1-AT and also of the soluble fractions of the expressed protein by SDS-PAGE. The overall expression level will represent how well the gene is transcribed and translated in *Escherichia coli,* whereas the soluble fraction measures how efficiently the recombinant protein folds into a normal conformation.

**FIGURE 2.**
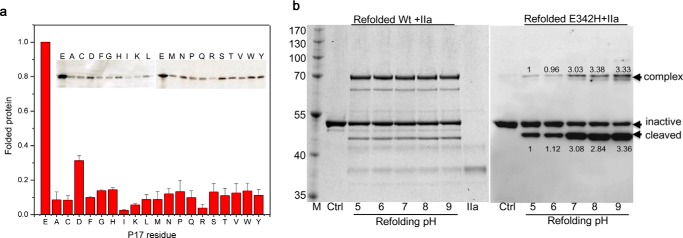
**Effect of residue 342 on folding of α1-AT.** *a,* 20 α1-AT variants with different residues at position 342 were expressed in *E. coli,* and the soluble fractions of the expressed α1-AT were analyzed by SDS-PAGE and Western blotting. The *inset* gel is shown as an example. The relative expression levels derived from densitometry analysis of four independent experiments were plotted with the wild type as mean ± S.D. The expression levels of all the mutants are significantly different from that of wild type α1-AT with *p* value < 0.05 as determined by Student's *t* tests. *b,* pH effects on the refolding of WT and E342H α1-AT. Denatured α1-AT was quickly diluted into refolding buffer of different pH values. The refolded α1-AT samples were then mixed with excess thrombin (IIa) and assessed for α1-AT·thrombin complex formation. The samples from wild type α1-AT refolding were analyzed by SDS-PAGE and Coomassie Blue staining (*left gel*), and samples for E342H were analyzed by Western blot (*right gel*) using anti-α1-AT antibody. The comparative result of densitometry analysis for complex or cleaved bands was shown in the gel, and the quantity of complex or cleaved α1-AT formation at pH 5 was set to 1.

As expected, we found that the overall expression level of all these α1-AT variants were similar, indicating that the mutations have little effect on α1-AT gene transcription and translation. Therefore, the soluble fraction of the expressed α1-AT for each variant will represent the variant's folding efficiency. The soluble fractions for all the variants were analyzed by SDS-PAGE and Western blotting (Fig. 2*a*). This revealed that all 19 mutations caused a significant reduction (∼70–90%) in the expression of soluble α1-AT as compared with the wild type. This is consistent with the notion that Glu-342 is critical for the folding of α1-AT. Among the mutants, E342D mutant has a lesser reduction (∼70%), likely due to partial preservation of stabilizing interactions by the chemically similar Asp side chain. Notably, soluble fractions from mutants where Glu-342 is mutated to residues with smaller side chains such as Ala and Gly are decreased to ∼10% of wild type α1-AT, and Arg and Lys mutations seem to have an even greater decrease with the soluble fractions being only ∼5% of the wild type.

As this aggravating effect of Arg and Lys likely arises from their positively charged side chains, we performed refolding experiments to test the effect on folding of charges at position 342. The denatured α1-AT E342H mutant was diluted into refolding buffer of different pH values, and the inhibitory activity of the folded α1-AT was assessed by SDS-PAGE. As shown in Fig. 2*b*, the correctly folded wild type α1-AT readily formed a stable complex with protease, with similar amounts being formed over a pH range of 5–9. This shows that refolding of wild type α 1-AT is not affected by changes of pH. However, pH changes have a significant effect on folding of E342H with a much lower yield at pH 5 and a higher yield at pH 9 (Fig. 2*b*, *right gel*). The increased amount of cleaved α1-AT E342H is proportional to the amount of complexes formed as indicated by densitometry analysis of the bands, which is consistent with the increased SI of Glu-342 variants and substrate behavior of misfolded proteins (29). As the histidine side chain is protonated around pH 5 and therefore positively charged, and is largely uncharged when the pH is above 8, this indicates that a positive charge at position 342 has an aggravating effect on the folding of α1-AT. Overall, the finding here indicates that the loss of the Glu-342 side chain, as seen with the E342A mutant, results in a significant (∼10-fold) decrease in folded α1-AT, although a positive charge at position 342 aggravates the folding process further.

##### Role of Residue 342 in α1-AT Activities

**FIGURE 3.**
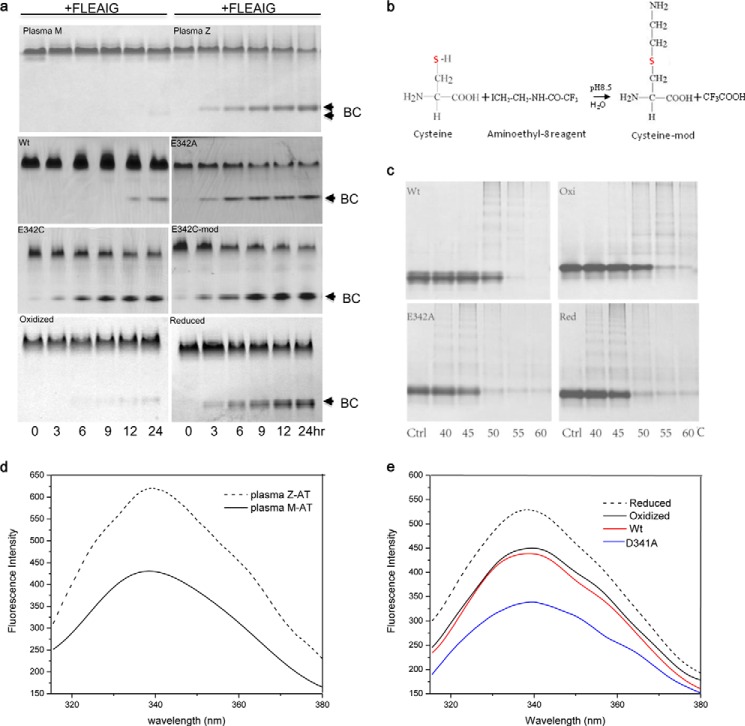
**α1-AT conformation probed by peptide annealing experiment (*a*), chemical modification (*b*), thermal stability assay (*c*), and fluorescent measurements (*d* and *e*).** *a,* α1-AT variants (0.5 mg/ml) were incubated with 100-fold excess 6-mer (FLEAIG) peptide at 37 °C for different time intervals and then analyzed by a native gel containing 7 m urea. The α1-AT·peptide binary complex (*BC*, *arrows*) is hyperstable and resistant to urea denaturation and migrates faster than the unfolded α1-AT. Plasma Z α1-AT, recombinant E342A, E342C, and E342C-mod α1-AT variants form a binary complex faster than plasma M α1-AT and recombinant wild type (*Wt*). The oxidized form of α1-AT mutant (S292C, T339C, and E342A, termed α_1_-AT-SS-E342A), where s5A is fixed to s6A by a disulfide bridge (*Oxi*), binds to 6-mer peptide slowly; however, its reduced form (*red*) readily binds to 6-mer peptide similar to other Glu-342 mutants. *b,* aminoethyl-8 reagent specifically reacts with thiol group and converts the cysteine side chain to a lysine-like structure. *c,* α1-AT variants were incubated at various temperatures in 10 mm Tris-HCl, pH 7.4, 0.1 m NaCl, and 0.5 m urea for 30 min and then analyzed by native gel electrophoresis. E342A mutant (E342A) has reduced thermal stability as compared with wild type α1-AT (*Wt*). The oxidized α1-AT mutant (*Oxi*) has similar thermal stability as that of the WT, but it becomes less stable once the disulfide is reduced (*red*). *d* and *e,* fluorescence emission spectra of α1-AT variants were measured at excitation wavelength 290 nm. The spectra of plasma M α1-AT is shown as a *black solid line* and Z α1-AT as a *dashed line* (*d*). The reduced form of the mutant α_1_-AT-SS-E342A is shown as a *black dashed line,* and the oxidized form as a *black solid line*. The spectra of the D341A variant and the wild type are shown in *blue* and *red solid lines*, respectively (*e*).

Subsequently, we attempted to purify and characterize the recombinant Z α1-AT (E342K) variant from the soluble fraction of *E. coli,* but we failed to get sufficient protein for further study. So we took an alternative approach by purifying a E342C mutant and then converting Cys-342 to a lysine-like residue *in vitro* by chemical modification (Fig. 3). The modified E342C variant, termed E342C-mod, was verified by mass spectrometry analysis. We then tested the inhibitory activities of the E342C, E342C-mod, and wild type α1-AT variants against thrombin and activated factor X (FXa). As shown in Table 1, all the mutants have nearly doubled stoichiometry of inhibition (SI) toward thrombin and FXa when compared with the wild type. Notably, the E342C mutant has an almost identical association rate (*k_a_*) and stoichiometric inhibition (SI) to those of E342C-mod in inhibiting thrombin, with the two mutants inhibiting thrombin more than 2.5-fold faster than the wild type protein. E342C mutant has a similar SI (3.0) as that of E342C-mod toward FXa. Therefore, the Z-like α1-AT mutant is still a good protease inhibitor but is less efficient as it requires about 2–3 molecules of inhibitor, instead of 1 with wild type α1-AT, to inhibit one protease molecule. This is consistent with previous observations that Z α1-AT derived from human plasma or recombinant Z α1-AT is largely active in inhibiting protease, although with increased SIs (30–32). Also, these data indicate that, once the α1-AT mutant is folded, the positive charge at residue 342 has little effect on the inhibitory activity of α1-AT because both E342C and E342C-mod behave similarly toward protease (Table 1).

**TABLE 1:** **Inhibition parameters (*K_a_* and SI) for α1-AT variants toward thrombin and activated factor X (FXa)** All the variants are based on α1-AT Pittsburgh backbone with (M358R and C232S). Each value was the average of three independent measurements with standard error shown.

	Thrombin			FXa		
	*K_a_*	SI	*K_a_*·SI	*K_a_*	SI	*K_a_*·SI
	*m*^−*1*^ *s*^−*1*^		*m*^−*1*^ *s*^−*1*^	*m*^−*1*^ *s*^−*1*^		*m*^−*1*^ *s*^−*1*^
Wild type	1.1 ± 0.1e5	1.6 ± 0.1	1.1e5	3.6 ± 0.1e4	1.3 ± 0.1	4.7e4
E342C	0.93 ± 0.1e5	3.0 ± 0.1	2.8e5	2.3 ± 0.1e4	3.0 ± 0.1	6.9e4
E342C-mod	0.88 ± 0.1e5	3.4 ± 0.2	3.0e5	1.2 ± 0.1e4	3.4 ± 0.1	4.1e4

##### Role of Residue 342 in α1-AT Conformations

To test the effect of these mutations at position 342 on α1-AT conformations, we performed a peptide annealing experiment in which Glu-342 variants were mixed with the 6-mer peptide FLEAIG, derived from P7 to P2 sequence of α1-AT reactive loop, and assessed for binary complex formation. It has been shown that peptides encoding the reactive center loop sequence could insert into the central β-sheet A as strand 4 (Fig. 1*c*) and convert a serpin into a hyperstable conformation (33, 34), which is resistant to denaturation in 7 m urea. As shown in Fig. 3*a*, plasma-derived Z α1-AT forms the α1-AT peptide hyperstable binary complex much faster than plasma-derived normal M α1-AT, which is consistent with previous observations (23, 35). As expected, E342C-mod readily forms a urea stable binary complex as seen with plasma-derived Z α1-AT. However, E342A and E342C mutants similarly form a binary complex with the 6-mer peptide much faster than wild type α1-AT (Fig. 3*a*). This suggests that all the Glu-342 mutants, regardless of the side chain of residue 342, can adopt an aberrant conformation, similar to that of plasma Z α1-AT, which binds the 6-mer peptide preferentially. Therefore, we conclude that the aberrant conformation of α1-AT arises from the loss of the stabilizing interactions of Glu-342 in wild type α1-AT, rather than from the positive charge of Lys in the Z variant.

##### Stabilization of s5A by a Disulfide Bridge

Because Glu-342 is located at the top of s5A and its key interaction is to anchor s5A to s6A, we engineered an alternative stabilizing interaction through a disulfide bond linking s5A and s6A (Fig. 1*a*) as described previously (16). This disulfide bond was used to probe intermolecular linkage between s5A and s6A (16), and here we tested its effect on the aberrant conformational change of Z α1-AT. These mutations (S292C and T339C) were introduced onto a E342A α1-AT background. Both the oxidized and reduced forms of this mutant (α1-AT-S292C-T339C-E342A, termed α1-AT-SS-E342A) were prepared and assessed in the peptide annealing experiment. As expected, under reducing conditions this mutant preferentially binds to the 6-mer peptide, as does the unmodified E342A mutant (Fig. 3*a*). The oxidized form, however, where s5A is fixed by a disulfide bond, behaves like wild type α1-AT, with slower insertion of the 6-mer peptide.

Notably, the oxidized form has a thermal stability similar to that of the wild type, being largely stable at 50 °C, but it becomes less stable under reducing conditions and is prone to form aggregates and polymers at 50 °C as does the E342A variant (Fig. 3*c*). This is in line with previous findings that plasma-derived Z α1-AT is less stable than normal M α1-AT and forms polymer after incubation at 37 °C or slightly elevated temperature (30).

Furthermore, fluorescence spectroscopic measurements showed that E342A mutant has a higher basal fluorescence signal than the wild type, which resembles those of plasma-derived normal M α1-AT and Z α1-AT (Fig. 3, *d* and *e*). To test whether formation of the disulfide bond in mutant α1-AT-SS-E342A induces any conformational change, the spectra of reduced or oxidized forms of this mutant were measured (Fig. 3*e*). The spectrum of the oxidized form resembles that of wild type α1-AT; however, once the disulfide is reduced, there is a substantial increase in the fluorescence intensity at 340 nm (Fig. 3*e*). These spectra are consistent with previous suggestions that Trp-194 (Figs. 1*a* and 3*d*), which is buried in the hinge region and forms hydrophobic interactions with Met-242 and Tyr-244 of s2/3B in the wild type α1-AT, becomes more solvent accessible in the Z conformation (31, 36). Therefore, we conclude that Z α1-AT has an unstable central β-sheet A, which allows preferential insertion of the 6-mer peptide, and the top half of s5A is labile with Trp-194 readily exposed.

##### Probing the Aberrant Conformation through mPEG Modification

**FIGURE 4.**
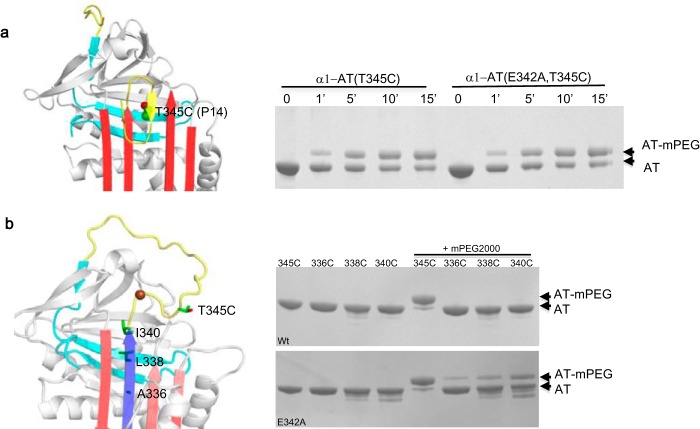
**Probing the aberrant conformation through mPEG modification.** *a,* accessibilities of cysteine residue at P14 position. α1-AT (T345C) and α1-AT (E342A/T345C) were mixed with mPEG, a specific cysteine alkylation reagent for various time intervals, and then the reaction was followed by SDS-PAGE analysis. Each modification would result in 2000Da increase in molecular weight of α 1-AT. *b,* accessibilities of residues of s5A in α1-AT. α1-AT variants based on the wild type or E342A backbones with residues Ile-340, Leu-338, Ala-336, and Thr-345 mutated to cysteine, respectively, were reacted with mPEG. AT-mPEG indicates the modified α1-AT. The positions of the selected residues in α1-AT structure are shown on the *left*. The models on the *left* were based on α1-antichymotrypsin δ form (43) in *a* and α 1-AT in *b*.

It was proposed that Z α1-AT might have a partially open β-sheet A with the reactive loop partially inserted (Fig. 4*b*), similar to the δ-conformation of antichymotrypsin (37). This would allow ready insertion of a short peptide, such as FLEAIG, into the lower half of β-sheet A, but not the longer peptide encoding P14-P3 of the reactive center loop (23). To test this hypothesis, we prepared two mutants with the P14 (Thr-345) residue replaced by Cys (Fig. 4*a*), on the backbone of both wild type and E342A α1-AT. Similarly, mutants with residues Ile-340, Leu-338, and Ala-336 around the top part of s5A mutated to cysteine, respectively, were prepared. If these residues become solvent-exposed or buried due to conformation change, they should have different accessibility toward modification. The variants were mixed with mPEG, a specific cysteine alkylation reagent. Each modification will result in a 2000-Da increase in molecular mass. The reaction was followed by SDS-PAGE analysis. Both mutants with P14 cysteine can be readily modified by mPEG (Fig. 4*a*) at a similar rate, indicating that the P14 residue is similarly surface-exposed in both wild type α1-AT and E342A variant. The residues (336, 338, and 340) with side chains pointing toward the hydrophobic core of the protein on the wild type α1-AT backbone (Fig. 4*b*, *left*) are resistant to modification as expected. Remarkably, residues on top of s5A on E342A backbone are accessible for mPEG modification (Fig. 4*b*, *lower gel*). This further confirms that the top half of s5A becomes labile once Glu-342 is mutated as indicated by the disulfide linkage experiment shown above. Also, the cysteine modification experiment (Fig. 4) together with fluorescence measurements (Fig. 3*c*) excludes the possibility that the reactive center loop of Z α1-AT would stay partially inserted (23).

##### Crystal Structure of Z α1-AT

**FIGURE 5.**
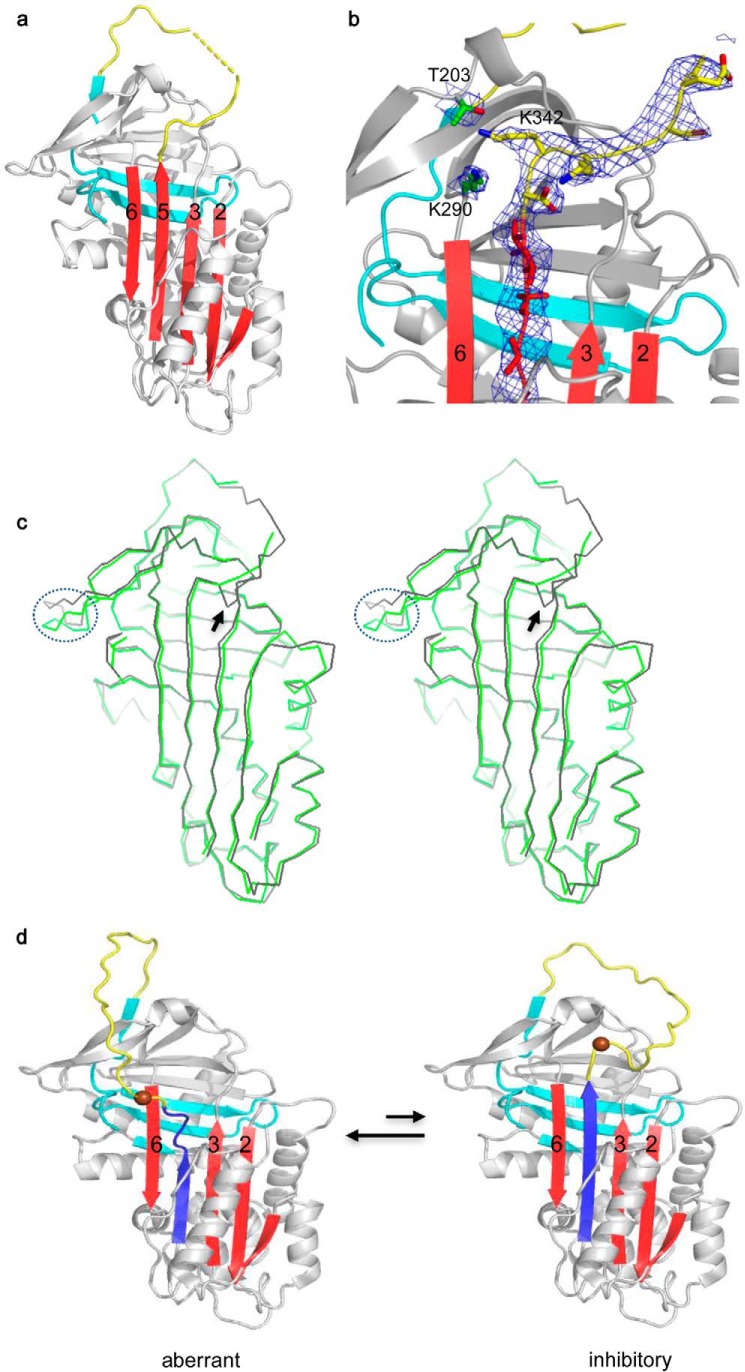
**Structures of Z α1-AT.** *a,* crystal structure of glycosylated Z α1-AT variant (M358R, C232S, and E342K) shows that Z α1-AT has a typical inhibitory fold as seen with other native serpins with a closed central sheet A (*red*) and a surface-exposed reactive loop (*yellow*). The C-terminal portion is colored in *cyan. b,* electron density map contoured at 1.0 σ around the hinge region shows an extended reactive loop in Z α1-AT. Residues 342, Thr-203, and Lys-290 occupy similar positions as seen in wild type. *c,* stereo view of overlaid structures of wild type (Protein Data Bank code 1QLP, *black*) and Z α1-AT (*green*). There are only subtle changes around the hinge region (*arrow*) and the gate region (*circled dashed line*) in Z α1-AT when compared with wild type α1-antitrypsin. *d,* simplified model of Z α1-AT conformations. Because of the loss of stabilizing interactions at position 342, the s5A (*blue*) of Z α1-AT is labile and equilibrates between partially and fully inserted conformations. It is likely the predominant conformation of Z α1-AT has a partially inserted s5A (*left*, aberrant conformation), which could readily form a binding site, with s3A, for the 6-mer peptide. Carbon α of residue 342 is shown as a *brown sphere*.

To further understand the conformation of Z α1-AT, we carried out crystallization screens on various α1-AT mutants expressed in *E. coli* and on glycosylated Z α1-AT expressed in *Drosophila* cells. Only crystals from glycosylated recombinant Z α1-AT yielded a good diffraction data set at 3.3 Å, and the structure was solved by molecular replacement (Table 2). Unexpectedly, the overall structure of Z α1-AT largely resembles that of wild type with a closed 5-stranded β-sheet A and a fully exposed reactive center loop (Fig. 5). Although the overall resolution of the structure is modest, the electron density map around Lys-342 is unambiguous as shown in Fig. 5*b*. It appears that the positively charged side chain has a minimal effect on overall packing around the hinge region of Z α1-AT. Nevertheless, superposition of wild type α1-AT with that of Z α1-AT reveals subtle differences between Z and wild type α1-AT, with a change in orientation of the reactive loop and a slight shift of s5A at the top half of β-sheet A (Fig. 5*c*). The reactive loop of Z α1-AT is fully extended, and in the wild type α1-AT structure and most other inhibitory serpins the reactive center loop characteristically takes a U turn around P17 and P16 with residues P15 and P14 poised to enter the central β-sheet (Fig. 1). Close inspection of crystal packing reveals that the P13 and P12 residues of the reactive loop from one Z α1-AT molecule are packed against either hH or hE from a symmetry-related molecule. It is likely that both the crystal packing and the increased flexibility around s5A allow Z α1-AT to crystallize in a native-like conformation with a relatively extended reactive loop.

**TABLE 2:** **Crystallographic data collection and refinement statistics**

Parameter	Value
**Data collection**	
Space group	P21
Cell dimensions	
(Å)	*a, b, c* = 74.13, 53.67, 110.14
(°)	α, β, γ = 90, 96.88, 90
Solvent content (%)	44
Wavelength (Å)	0.9763
Resolution (Å)	74.54–3.3; 3.48–3.3
Total reflections	55,690; 7261
Unique reflections	12,855; 1803
Multiplicity	4.3; 4.0
Mean *I*/S.D.(*I*)	7.7; 1.5
Completeness (%)	98.9; 96.1
*R*merge	0.136; 0.946

**Refinement**	
No. of atoms modeled	5836
Protein	5806
Water	2
Heterogen	28
Average *B*-factor (Å^2^)	82.96
Reflections in working/free set	11,967/642
*R*-factor/*R*-free (%)	0.22/0.28
r.m.s.*^a^* deviation of bonds (Å)/ angles (°)	0.004/0.9
Ramachandran plot (favored/outliers, %)	95.42/0
MolProbity score	1.54, 100th percentile*^b^* (*n* = 865, 3.34 ± 0.25 Å)

*^a^* r.m.s. is root mean square.

*^b^* 100th percentile is the best among structures of comparable resolution; 0th percentile is the worst.

Our findings here suggest that the aberrant conformation of Z α1-AT results from a labile s5A and that stabilization of s5A would lead to a wild type like conformation. As Z α1-AT is largely active, it must have the ability to adopt a normal inhibitory conformation for protease inhibition as seen in its crystal structure shown here. This structure of Z α1-AT allows us to propose that Z α1-AT has a labile s5A and equilibrates between a native inhibitory conformation as shown in its crystal structure and an aberrant conformation with s5A only partially incorporated into the central β-sheet (Fig. 5*d*). This is consistent with previous predications that Z α1-AT has an increased flexibility around the hinge region (21, 31, 36, 38).

##### Binding of PBA on α1-AT

**FIGURE 6.**
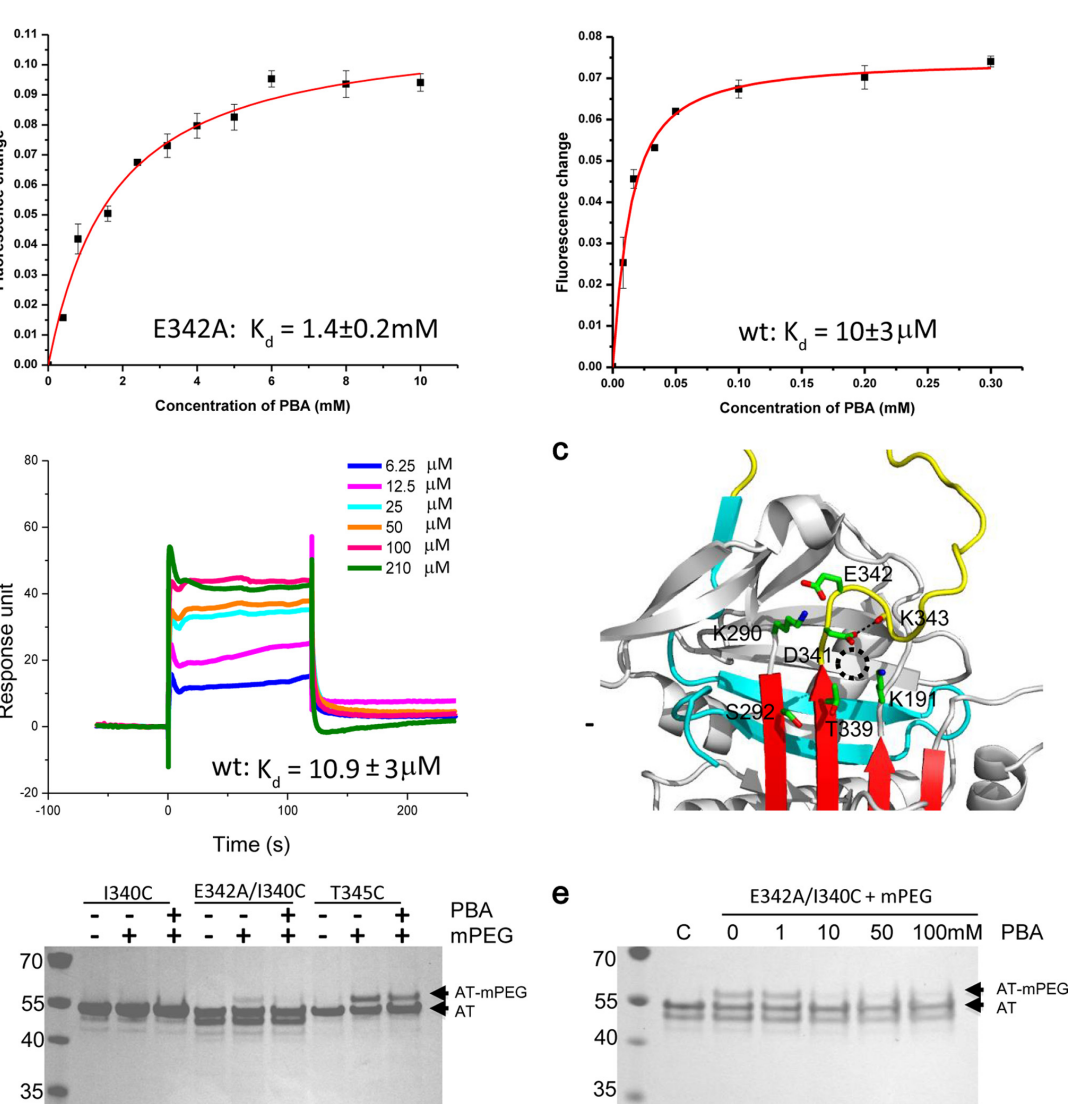
**Binding of PBA on α1-AT.** *a,* binding affinities of α1-AT E342A mutant and WT α1-AT were measured by fluorescent titration with estimated *K_d_* values of 1.4 ± 0.2 mm for E342A mutant (*n* = 3) and 0.010 ± 0.003 mm for WT (*n* = 3), respectively. *b,* BIAcore diagram of PBA binding to WT α1-AT showed that PBA rapidly associated and dissociated from the immobilized α1-AT. *K_d_* value was estimated from a steady-state affinity model by BIAcore T200 analysis software. *c,* residues around the hinge region were mutated in α1-AT and assessed for PBA binding. The corresponding *K_d_* values are listed in Table 3. The breach region for the reactive loop to insert is *circled* by *dashed lines. d,* PBA effect on the accessibility of cysteine residues in α1-AT variants was assessed by mPEG modification where α_1_-AT variants (I340C, E342A/I340C, and T345C) were mixed with 20 μm mPEG in the presence or absence of 100 mm PBA at room temperature for 10 min before being analyzed by SDS-PAGE. *e,* concentration effect of PBA on modification of E342A/I340C mutant by mPEG was similarly assessed in the presence of different concentrations of PBA. *AT-mPEG*, mPEG modified α 1-AT.

It has been shown previously that small molecules, chemical chaperones such as PBA, could stimulate Z α1-AT secretion from cells. More importantly, PBA was shown to increase the concentration of Z α1-AT in circulation in a PiZZ mouse model (39). PBA is a Food and Drug Administration-approved drug for treatment of urea cycle disorder in humans; however, it is not well understood how this compound functions on the Z α1-AT folding pathway. Here, we tested whether PBA could directly bind to Z α1-AT. Fluorescence titration measurements showed that PBA did bind to plasma Z α1-AT, but very weakly with a dissociation constant (*K_d_*) of 1.23 mm. We further assessed the binding affinity of plasma M α1-AT and recombinant wild type α1-AT. They had a similar binding affinity to PBA with a *K_d_* of 0.011 and 0.010 mm respectively. In contrast, recombinant E342A mutant bound to PBA very weakly with a *K_d_* of 1.4 mm, similar to that of Z α1-AT (Table 3 and Fig. 6*a*). To validate the *K_d_* values measured by fluorescence titration, we also applied the surface plasmon resonance experiments. The sensorgrams showed that PBA rapidly associated and dissociated from the immobilized α1-AT (Fig. 6*b*); therefore, the *K_d_* values could only be estimated from a steady-state affinity model due to the fast *k*_on_ and *k*_off_ values (40). Wild type α1-AT and K191A mutant bound PBA with *K_d_* values of 0.005 and 0.02 mm, respectively, consistent with the values measured by fluorescence titration. The *K_d_* value for E342A mutant binding of PBA measured by surface plasmon resonance was 0.1 mm, which was much smaller than the value measured by fluorescence titration (Table 3). This might be due to changes in α1-AT flexibility once protein was immobilized onto a surface. Therefore, these measurements confirmed that E342A and Z α1-AT mutants bound PBA significantly weaker than wild type α1-AT.

**TABLE 3:** ***K_d_* values for α1-AT variants binding of PBA measured by fluorescence titration and surface plasmon resonance assays** All the variants are based on α1-AT Pittsburgh backbone with (M358R and C232S). The oxidized form of α1-AT-SS-E342A is termed Oxi here.

	Fluorescence *K_d_*	Surface plasmon resonance *K_d_*
	*mm*	*mm*
WT	0.010 ± 0.003	0.005 ± 0.001
E342A	1.4 ± 0.2	0.100 ± 0.025
K191A	0.019 ± 0.006	0.020 ± 0.003
T339A	0.015 ± 0.002	
K290A	1.61 ± 0.30	
D341A	1.38 ± 0.20	
Oxi	0.016 ± 0.005	
Oxi -K290A	0.017 ± 0.007	
M α1-AT	0.011 ± 0.004	
Z α1-AT	1.23 ± 0.14	

To assess whether this increase in *K_d_* values of these mutants is associated with the increased flexibility of s5A, we measured the binding affinity of α1-AT-SS-E342A mutant with s5A and s6A linked by a disulfide bridge as described above. When the disulfide was formed, the mutant bound PBA with a *K_d_* of 0.016 mm similar to that of wild type α1-AT (Table 3). This suggests that the side chain of Glu-342 is not directly involved in binding PBA, and the high binding affinity of α1-AT critically depends on the stability of s5A. Therefore, we reasoned that the PBA-binding site on α1-AT is likely located near the top part of s5A. Several residues such as Lys-191, Lys-290, Thr-339, and Asp-341 around the top part of s5A (Fig. 6*c*) were selected and mutated to Ala with corresponding variants assessed for PBA binding. We found that K191A and T339A mutants had a modest decrease in binding affinity with a *K_d_* of 0.019 and 0.015 mm toward PBA, respectively. K290A mutant bound PBA with a *K_d_* of 1.61 mm, similar to that of Z α1-AT (Table 3). Because Lys-290 is involved in forming a highly conserved stabilizing interaction with Glu-342 (Fig. 1*a*), this decreased binding affinity arose either from the labile s5A following the loss of its stabilizing interactions or from the loss of the direct interaction between Lys-290 and PBA. So we introduced K290A into α1-AT-SS-E342A backbone and measured this mutant's binding affinity. The oxidized form of this mutant (α1-AT-SS-E342A-K290A) behaved the same as α1-AT-SS-E342A with a *K_d_* of 0.017 mm. Therefore, this further confirms that the stabilization interactions between Glu-342 and Lys-290 and the stability of s5A are critical for high affinity PBA binding of α1-AT, and Lys-290 does not directly interact with PBA.

Remarkably, the D341A mutant had normal activity in protease inhibition, but it had a lower basal fluorescence signal than wild type α1-AT (Fig. 3*e*), and bound PBA weakly with a *K_d_* value the same as that of α1-AT-E342A mutant (Table 3). By examining the crystal structures of α1-AT in the Protein Data Bank, we found that Asp-341 was either solvent-exposed, similar to that in Z α1-AT structure shown here, or formed hydrogen bonds with the main chain oxygen atom of residue 343 maintaining a U turn of the reactive loop in the hinge region (Fig. 6*c*). Therefore, the decreased basal fluorescence signal of D341A (Fig. 3*e*) likely arises from the fully extended reactive loop that affects the local environment of Trp-194 below the β-sheet A. Altogether, these data suggest that Asp-341 on the top of s5A plays a key role in maintaining the high affinity PBA-binding site in α1-AT, and other residues such as Lys-191, Lys-290, Ser-292, Thr-339, and Glu-342 play a minor role. We speculate that PBA likely binds α1-AT near the breach region in α1-AT (Fig. 6).

##### Effect of PBA on the Aberrant Conformation of Z α1-AT

Because PBA could bind α1-AT near the top part of s5A, we further checked whether its binding would have any effect on the aberrant conformation of Z α1-AT, particularly on the stability of s5A. The accessibility of residue 340 in the α1-AT-I340C and α1-AT-E342A-I340C was similarly assessed by mPEG modification in the presence or absence of PBA. As shown in Fig. 6*d*, modification of I340C (*lanes 2* and *3*) and T345C (*lanes 8* and *9*) mutants were unaffected by PBA (Fig. 6*d*) consistent with Fig. 4*b* above. However, in the absence of PBA the I340C/E342A mutant could be modified by mPEG, and it became resistant to modification in the presence of PBA (Fig. 6*d*). Furthermore, the effect of PBA on I340C modification by mPEG was concentration-dependent (Fig. 6*e*). PBA was more effective in protecting residue 340 from modification when its concentration was >10 mm. Therefore, we conclude that PBA binds to Z α1-AT and stabilizes s5A.

## Discussion

### 

#### 

##### Aberrant Conformation of Z α1-AT

It has long been proposed by several groups that Z α1-AT likely exists in an abnormal conformation (7, 23, 30, 41, 42). The plasma-derived Z α1-AT is largely active, but polymerogenic, with higher basal fluorescence intensity than normal M and preferential binding of a 6-mer peptide. Our studies here are consistent with these previous observations and have further revealed that Z mutation results in the loss of Glu-342 interactions and consequently a labile s5A in Z α1-AT. This aberrant conformation readily explains why Z α1-AT preferentially binds a 6-mer peptide at the bottom half of β-sheet A. Also, a labile s5A will weaken the packing below the β-sheet A of Z α1-AT with consequent exposure of Trp-194 (Fig. 3) leading to polymer formation of Z α1-AT during incubation at elevated temperature.

##### Labile s5A and Mechanism of Z α1-AT Polymerization

As *in vivo* polymerization of α1-AT caused by Z mutation occurs mainly in the endoplasmic reticulum, this suggests that folding intermediates, not the native state, play the dominant role in polymerization. The mechanism underlying the pathological Z α1-AT deficiency will be centered on the following two interlinked questions. How is α1-AT folded into a metastable conformation and how does the Z mutation disrupt the folding pathway and lead to polymerization? Understanding the folding defects of Z α1-AT is critical for selecting and designing reagents to rectify the Z α1-AT folding process. As we have shown here, the Z mutation will disrupt the packing of central β-sheet A and lead to a labile s5A. We believe that the same defect would perturb a key step in the α1-AT folding pathway and result in the pathological polymerization of Z α1-AT.

**FIGURE 7.**
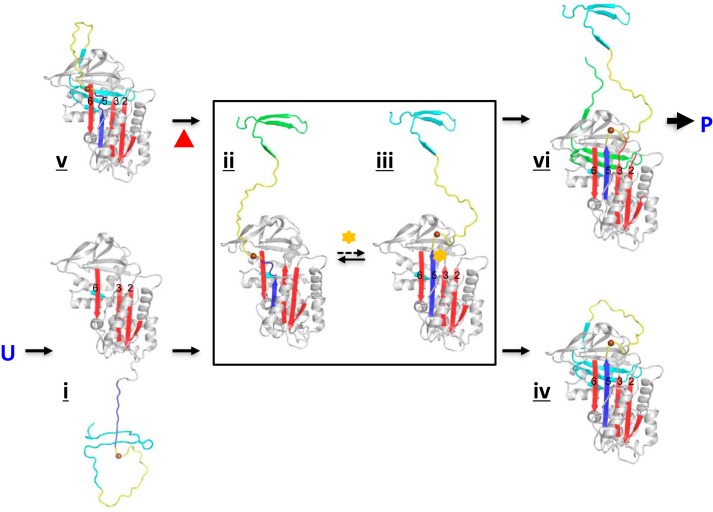
**Role of s5A in the folding and polymerization pathway of α1-AT.** When the nascent α1-AT polypeptide chain is synthesized, its N-terminal portion is first folded into a molten globule-like conformation with incomplete β-sheets A (*red*) and B and the unstructured C-terminal portion (*i*). Normally, the next step of folding is the association of s5A (*iii*), not s4/5B (21). Once the native-like 5-strand conformation of β-sheet A (*iii*) has been completed, the C-terminal portion, including the reactive center loop (*yellow*) and strands 1C, s4/5B (*cyan* or *green*) then starts to associate and form, thus forming the metastable conformation (*iv*). Because of mutation at 342 (*brown sphere*), the s5A (*blue*) of Z α1-AT could not stably anneal into the central β-sheet A leading to accumulation of an intermediate conformation with a partially inserted s5A (*ii*). Subsequent inter-molecular insertion of the C-terminal portion (*vi*) and rearrangement of the reactive loop lead to hyper-stable serpin polymer (*P*) formation. When folded Z α1-AT (*v*) is incubated at elevated temperature, the C-terminal portion becomes unstable and is released due to weakened packing between β-sheet A and B (*ii*). Subsequently, the same inter-molecular linkage could occur. Once the reactive loop (*yellow*) from the interlinked molecules (*vi*) starts to insert into the central β-sheet A as in Fig. 1*b*, the whole process become irreversible leading to serpin polymer formation. Small compounds such as PBA (*yellow star*) may bind and stabilize s5A in the intermediate conformation (*iii*) during the folding pathway promoting native metastable conformation formation (*iv*). Although s6A is likely disordered in the absence of s5A (39), it is shown here as a β-strand for simplicity.

Our findings here fit best with the sequential folding pathway proposed by Dolmer and Gettins (21) and with the crystal structure of α1-AT trimer solved by the Huntington and co-workers (17). In this folding model (Fig. 7), the N-terminal portion of a serpin is first folded into a molten globule-like conformation with incomplete β-sheets A and B and an unstructured C-terminal portion (Fig. 7*i*). The next step of folding is the association of s5A (Fig. 7*iii, blue*), not s4/5B. Once the native-like five-stranded conformation of β-sheet A has been completed (Fig. 7*iii*), the C-terminal portion, including the reactive loop (which would become s4A in the hyperstable state) and strand s1C and s4/5B, then starts to associate. It is through this ordered folding process that the serpin avoids a hyperstable conformation and folds into a metastable native state (Fig. 7*iv*) (21).

It is clear from this folding pathway how Z α1-AT would form polymers (21). As illustrated above (Fig. 7), annealing of s5A into the central β-sheet is critical for the subsequent folding steps. Loss of the key interactions caused by the Z mutation will result in an intermediate conformation (Fig. 7*ii*) with a partially inserted s5A. Because s5A of Z α1-AT could transiently fully incorporate into the β-sheet A (Fig. 7*iii*), as confirmed by its inhibitory activity and the crystal structure here, a small amount of Z α1-AT could be folded, with a properly inserted s4/5B (Fig. 7*iv*), and hence be secreted. This explains why there is ∼15% of active Z α1-AT monomer present in the circulation. However, the majority of this intermediate conformation would accumulate due to the delayed folding process caused by the Z mutation and will be prone to intermolecular insertion of s4/5B (Fig. 7*vi*). This will lead to polymer formation consistent with the crystal structure of an α1-AT trimer (Fig. 1*e*) where polymers are formed though a C-terminal s4/5B domain swap mechanism (17). Similarly, other mutations around the shutter region of α1-AT affecting either the annealing of s5A or subsequent insertion of C-terminal s1C and s4/5B would slow down the formation of the native monomer, predisposing to intermolecular linkage and subsequent polymer formation (Fig. 7).

When Z α1-AT derived from plasma, folded but with a labile conformation (Fig. 7*v*), is heated *in vitro*, its C-terminal portion, including the reactive center loop (*yellow*) and strands 1C, s4/5B (*cyan* or *green*), will predictably be readily detached from the main body of the molecule due to weakened packing between β-sheet A and B. This intermediate conformation likely resembles those of the serpin folding pathway (Fig. 7, *ii* and *iii*) and is prone to intermolecular linkage. Once the reactive loop of the interlinked molecule (Fig. 7*vi*) inserts as a middle strand of the central β-sheet A, this process will be irreversible leading to hyperstable polymer formation (Fig. 1*e*).

##### Therapeutic Approach toward Z α1-AT Deficiency by Targeting Labile s5A

There have been numerous attempts to design and select reagents to prevent Z α1-AT polymerization (13, 39, 43, 44). So far, the most effective compound is PBA discovered by serendipity, which can increase Z α1-AT in PiZZ in mouse circulation (39). Although the chemical chaperones are often considered to function through nonspecific binding, some do directly interact with ligands or the protein active site (45, 46). Here, we have found that PBA could readily bind wild type α1-AT relatively tightly with a *K_d_* of 10 μm, but it binds Z α1-AT more than 120 times weaker (Fig. 6). Our mutagenesis study indicates that the binding site is located near the hinge region with residue Asp-341 playing a key role in maintaining the high binding affinity. More importantly, we show that PBA binding could stabilize the top half of s5A (Fig. 6). Although the physiological role of the specific interactions between PBA and Z α1-AT requires further investigation, we speculate that PBA could act similarly in stabilizing s5A during the folding pathway (Fig. 7*iii*) *in vivo* promoting the folding of monomeric Z α1-AT and subsequent secretion from the cells. Therefore, targeting labile s5A could be a viable approach toward Z α1-AT deficiency. The limited effect of PBA in human Z α1-AT patients (47) may arise from the poor binding affinity of PBA toward Z α1-AT and its severe side effects.

Overall, our data show here the Z mutation destabilizes the s5A of α1-AT leading to an aberrant conformation of Z α1-AT monomer. The same defect would disrupt a key step in α1-AT folding pathway leading to the pathological Z α1-AT polymerization via the C-terminal s4/5B domain swap mechanism (17, 21). Most importantly, our finding demonstrates that previously identified small molecule PBA, which partially ameliorates Z α1-AT deficiency in mice, may act on Z α1-AT by stabilizing s5A. This opens a potential therapeutic approach toward Z α1-AT deficiency by designing and selecting more effective agents through targeting the top half of s5A.

## Experimental Procedures

### 

#### 

##### Preparation and Characterization of α1-Antitrypsin Variants

Human α1-AT cDNA was amplified by polymerase chain reaction and inserted into the expression vector pQE31 as described previously (48). All the α1-AT variants were based on the α1-AT Pittsburgh backbone (M358R and C232S) and verified by DNA sequencing. The recombinant α1-AT was expressed with an MRSHHHHHH tag at the N terminus and purified from the soluble fraction of *E. coli* lysate. All the recombinant proteins were purified to homogeneity (>95% purity) and confirmed by SDS-PAGE. Modification of the E342C α1-AT mutant by aminoethyl-8 reagent (*N*-(iodoethyl)trifluoroacetamide, Pierce) was performed at pH 8.5 according to the manufacturer's instructions. The modified variant is termed E342C-mod and was verified by mass spectrometry. It migrates slower than the E342C variant in a native gel.

Recombinant glycosylated α1-AT was also prepared from *Drosophila* S2 cells using expression vector pMT/BiP/V5-His. Expression of the recombinant Z α1-AT (M358R, C232S, T345C, and E342K), where P1 residue Met-358 was mutated to Arg for convenient activity assessment and P14 residue Thr-345 was mutated to Cys for probing the conformation of the reactive loop around the hinge region, was induced by adding 0.5 mm copper sulfate into S2 cell culture at 23 °C. The secreted recombinant protein was purified from the medium using nickel-chelating Sepharose beads and subsequent HiTrap Q ion exchange chromatography. The behavior of the recombinant Z variant is similar to that of plasma-derived Z α1-AT in preferential binding of the 6-mer peptide (FLEAIG) (23). Plasma-derived Z α1-AT was provided by Dr. Helen Parfrey, University of Cambridge (35). Peptide insertion experiments were performed by incubating α1-AT variants (0.5 mg/ml) with 100-fold molar excess of peptides for different time intervals at 37 °C. Samples were then analyzed on an 8% (w/v) native gel with 7 m urea. Stoichiometries and rates of inhibition for the interaction between α1-AT and proteases were performed as described previously (48). Binding affinity of PBA toward α1-AT variants was measured by titrating PBA stock solution into α1-AT with the decreased fluorescence signal of α1-AT followed (49). Modification of free thiol group in cysteine by NEM-PEG2000 (mPEG) was performed as described previously (50).

##### Refolding of α1-AT

Small scale protein refolding assays were performed by placing a small droplet of denatured α1-AT Pittsburgh (termed wild type) or E342H mutant in 6 m guanidine HCl at the bottom of a microcentrifuge tube and quickly diluting the protein with buffers at different pH values (pH 5–9) containing 100 mm NaCl. For the activity assay, refolded α1-AT was directly mixed with excess thrombin and incubated at room temperature for 15 min and then analyzed by SDS-PAGE and/or Western blotting.

##### Binding Affinity Measurement

The affinity of PBA toward α1-AT variants was measured by the fluorescence titration method where stock solutions of PBA were gradually titrated into α1-AT in phosphate-buffered saline with the fluorescence signal of the protein recorded at the 340-nm wavelength. The dissociation constant of the binding (*K_d_*) was fitted with 1:1 binding mode by Origin software. The affinity of PBA toward α1-AT variants was also analyzed on a BIAcore T200 machine with CM7 chips (GE Healthcare). PBSP buffer (phosphate-buffered saline containing 0.005% P20) was used as the analysis buffer. α1-AT variants were immobilized on the chip through amine coupling chemistry, and PBA was diluted to concentrations ranging from 6.25 to 210 μm. PBA was flowed through the chip at a rate of 30 μl/min, and the response unit was measured. The sensor surface was regenerated with 10 mm glycine, pH 2.5, at the end of each cycle. Sensorgrams were fitted with BIAcore T200 analysis software using a 1:1 binding mode, and the *K_d_* values were calculated with a steady-state affinity model due to the fast *k*_on_ and *k*_off_ (40).

##### Crystallization of Z α1-AT and Data Collection and Refinement

Recombinant Z α1-AT (M358R, C232S, T345C, and E342K) derived from S2 cells was concentrated to 17.2 mg/ml in 10 mm Tris-HCl, 50 mm NaCl. Crystallization was performed using sitting drop methods where Z α1-AT was mixed with equal volumes of reservoir solution and equilibrated against 10–20% PEG 4000 in 50 mm sodium cacodylate buffer, pH 6.8, and 0.2 m NH_4_F, with or without 12% glycerol. Thin plate-like crystals grew to full size in 2 weeks. Diffraction data up to 3.3 Å were collected from a single frozen crystal and processed with Mosflm and Scala from the CCP4 suite (51, 52). The structure was solved by Phaser (53) using Protein Data Bank code 1QLP (13) as a search model, and refinement was performed with Refmac from the CCP4 suite. Processing and refinement statistics are summarized in Table 2. The final refined structure has two copies in the asymmetric unit. Residues 1–23 and 347–352 in molecule A and residues 1–23 and 348–354 in molecule B are unresolved. The coordinates and structure factors have been deposited in the Protein Data Bank (accession number 5IO1). Carbohydrates at glycosylation sites ASN46 were not built into the model due to relatively weak electron density. Figures were made using PyMOL (54).

## Author Contributions

A. Z. and G. C. designed all experiments. X. H., Y. Z., F. Z., Z. W., R. J. R., and A. Z. performed the experiments. All authors contributed to data analysis. X. H., Y. Z., R. W. C., R. J. R., and G. C. contributed to manuscript preparation, and A. Z. wrote the paper.

## Footnotes

4 The abbreviations used are:

α1-AT: α1-antitrypsin
PBA: 4-phenylbutyrate
s5A: strand 5 of the central β-sheet A
s4/5B: strand 4/5 of the β-sheet B
mPEG: NEM-PEG2000
FXa: activated factor X
SI: stoichiometric inhibition.
